# Low salivary cortisol levels in patients with rheumatoid arthritis exposed to oral glucocorticoids: a cross-sectional study set within UK electronic health records

**DOI:** 10.1136/rmdopen-2018-000700

**Published:** 2018-10-01

**Authors:** Rebecca M Joseph, David W Ray, Brian Keevil, Tjeerd P van Staa, William G Dixon

**Affiliations:** 1 Arthritis Research UK Centre for Epidemiology, Centre for Musculoskeletal Research, School of Biological Sciences, Manchester Academic Health Science Centre, The University of Manchester, Manchester, UK; 2 Division of Diabetes, Endocrinology and Gastroenterology, School of Medical Sciences, Manchester Academic Health Science Centre, The University of Manchester, Manchester, UK; 3 Department of Clinical Biochemistry, Wythenshawe Hospital, Manchester University NHS Foundation Trust, Manchester, UK; 4 Health eResearch Centre, Centre for Health Informatics, School of Health Sciences, Manchester Academic Health Science Centre, The University of Manchester, Manchester, UK; 5 Faculty of Science, Division of Pharmacoepidemiology and Clinical Pharmacology, Utrecht University, Utrecht, The Netherlands; 6 Rheumatology Department, Salford Royal NHS Foundation Trust, Salford, UK; 7 NIHR Manchester Biomedical Research Centre, Manchester University NHS Foundation Trust, Manchester Academic Health Science Centre, Manchester, UK

**Keywords:** HPA-axis, arthritis, rheumatoid, electronic health records, glucocorticoids, observational study, pharmacoepidemiology

## Abstract

**Background:**

Glucocorticoids (GCs) suppress endogenous cortisol levels which can lead to adrenal insufficiency (AI). The frequency of GC-induced AI remains unclear. In this cross-sectional study, low morning salivary cortisol (MSC) levels were used as a measure of adrenal function. The study aim was to investigate the prevalence of low MSC in patients with rheumatoid arthritis (RA) currently and formerly exposed to oral GCs, and the association with potential risk factors.

**Methods:**

Sample collection was nested within UK primary care electronic health records (from the Clinical Practice Research Datalink). Participants were patients with RA with at least one prescription for oral GCs in the past 2 years. Self-reported oral GC use was used to define current use and current dose; prescription data were used to define exposure duration. MSC was determined from saliva samples; 5 nmol/L was the cut-off for low MSC. The prevalence of low MSC was estimated, and logistic regression was used to assess the association with potential risk factors.

**Results:**

66% of 38 current and 11 % of 38 former GC users had low MSC. Among former users with low MSC, the longest time since GC withdrawal was 6 months. Current GC dose, age and RA duration were significantly associated with increased risk of low MSC.

**Conclusion:**

The prevalence of low MSC among current GC users is high, and MSC levels may remain suppressed for several months after GC withdrawal. Clinicians should therefore consider the risk of suppressed cortisol and remain vigilant for symptoms of AI following GC withdrawal.

**Key messages**

**What is already known about this subject?**

- Many patients with rheumatoid arthritis take oral glucocorticoids and may be at risk of adrenal insufficiency
- Existing literature is unclear regarding the prevalence of adrenal insufficiency during and after glucocorticoid exposure.

**What does this study add?**

- This study found 66% of current users and 11% of former glucocorticoid users had low morning salivary cortisol levels, a surrogate measure of adrenal suppression.
- Current dose was significantly associated with risk of low morning salivary cortisol.

**How might this impact on clinical practice?**

- The results suggest a high proportion of patients exposed to glucocorticoids may be at risk of adrenal insufficiency upon withdrawal of glucocorticoids
- Clinicians should be vigilant whenever glucocorticoids are withdrawn and for months thereafter.

## Introduction

Many patients with rheumatoid arthritis (RA) will be exposed to oral glucocorticoids (GCs) as part of their RA treatment. In UK primary care, around half of patients with RA are prescribed oral GCs^1^ and 13% of patients receive prescriptions of at least 3 months’ duration.^2^ However, GCs are associated with a range of serious and non-serious side effects,^3 4^ one of which is suppression of the hypothalamic–pituitary–adrenal (HPA) axis.

Suppression of the HPA axis and resulting adrenal insufficiency (AI) have been associated with exposure to exogenous GCs for almost as long as GCs have been in therapeutic use.^5 6^ Exogenous GCs act through the GC receptor to exert negative feedback on the HPA axis, repressing corticotropin-releasing hormone and adrenocorticotropic hormone (ACTH) and thus reducing cortisol production.^7–10^ If the HPA axis remains suppressed after withdrawal of exogenous GCs, the patient is in a state of AI.^9 11^ AI can result in a potentially fatal adrenal crisis if the patient is exposed to stress, such as intercurrent infection.^12^ Chronically low cortisol levels can impact quality of life, with typical symptoms including fatigue, low energy, muscle weakness, nausea, weight loss, and aches and pains.^10^

The risk of developing adrenal insufficiency following GC exposure remains unclear.^13 14^ It is also unclear whether the risk is associated with increasing dose and duration of GCs as the current literature is heterogeneous and contains conflicting results.^13–16^ In addition, other factors such as the compound identity, route of administration, indication and significant individual variation are likely to affect the risk of AI.^11 14^ It is therefore difficult to assess the risk of GC-induced AI for particular patients or patient groups based on the current literature.

Typically a confident diagnosis of adrenal insufficiency requires a dynamic test of HPA-axis function in which the cortisol response to a stressor is assessed. The insulin tolerance test (ITT) is the gold-standard diagnostic test, and the ACTH-stimulation test is widely used in practice to assess adrenal reserve.^17 18^ However, assessing cortisol levels in morning-collected saliva samples is a convenient alternative to collecting blood samples.^19 20^

The aim of this study was to investigate the impact of exposure to oral GCs on adrenal function as measured by morning salivary cortisol level. The number of patients with cortisol levels below the threshold of normal, and thus considered to be at risk of AI, was quantified in patients with RA currently and formerly exposed to oral GCs. The associations between potential risk factors, including dose and duration of GC exposure, and low salivary cortisol were investigated.

## Methods

### Design and setting

This was a cross-sectional study combining data from routinely collected electronic health records (EHRs) with data collected directly from participants. Full details of the study design and recruitment process have been described previously.^21^ In brief, participants were recruited through English general practices and completed the study on a single morning between October 2015 and April 2016 (the ‘study completion date’).

### Electronic health records

Primary care EHR were provided by the Clinical Practice Research Datalink^22^ (CPRD), a UK research database containing pseudonymised data from over 700 general practices. CPRD provided depersonalised EHR for all participants who consented to take part in the study. Although identifiable information was collected from participants in order to conduct the study, the data flow ensured that the depersonalised EHR could never be linked to identifiable data by either the study team or CPRD.^21^

### Participants and recruitment

Eligible participants were adult patients with rheumatoid arthritis (defined using a validated algorithm^23^), who had been prescribed oral GCs within 2 years of the study and were registered at a general practice taking part in the study. Participants were excluded if they had a medical condition known to affect adrenal function (see online supplementary file 1), or less than 2 years of data within CPRD. To recruit participants, the CPRD database was searched for potentially eligible patients. General practices were approached to take part, and those taking part screened the list of potentially eligible patients and sent invitations to screened patients. Interested patients then contacted the study team directly, completing a consent form if they were willing to take part.

10.1136/rmdopen-2018-000700.supp1 Supplementary data

### Data collection

Participants were mailed study packs containing a saliva collection kit (Salivette Cortisol; Sarstedt) and paper diary (online supplementary file 2). To collect the sample, participants chewed on the cotton swab for approximately 2 min. The sample was then returned by mail to the study team (cortisol levels in saliva are stable at room temperature for a number of days^24^) and stored frozen at maximum −20˚C until analysed. The samples were analysed for salivary cortisol and cortisone by tandem mass spectrometry (see section salivary cortisol and cortisone levels).

10.1136/rmdopen-2018-000700.supp2 Supplementary data

Participants were instructed to collect the saliva sample on a morning of their choice (ie, their study completion date) half an hour after waking, avoiding eating, drinking anything but water, smoking or brushing their teeth until they had collected their saliva sample. Participants were asked not to take any GC medication until after collecting the sample. Information about compliance with these instructions was collected in the first part of the study diary. The remainder of the diary collected information about recent use of oral GCs and date of the most recent GC injection (see section GC exposure for further details). Participants were asked to complete the diary after collecting their sample and then mail the diary back to the study team.

### Salivary cortisol and cortisone levels

To determine cortisol and cortisone concentrations, LC-MS/MS analysis for salivary cortisol/cortisone was performed using a Waters Xevo TQ-MSTM mass spectrometer and a Waters AcquityTM LC system with an electrospray source operated in positive ionisation mode,^25^ which gave lower limits of detection 0.80 nmol/L (salivary cortisol) and 0.50 nmol/L (salivary cortisone). Intra-assay Coefficient of Variations (CVs) were less than 9.3% and less than 7.9% and inter-assay CVs were less than 9.7% and less than 10.3% at 1.8–52.2 nmol/L of salivary cortisol and 3.6–96 nmol/L of salivary cortisone, respectively. Based on assay-specific and laboratory-specific reference ranges (ie, 95% CIs) established in healthy volunteers aged 25–55,^26^ the lower level of normal was 5 nmol/L for salivary cortisol and 18 nmol/L for salivary cortisone.

### GC exposure

GC exposure was defined using both data from the study diary and prescription data from CPRD. The following variables were defined: current use (exposed/unexposed) of oral GCs, current dose of oral GCs (mg, prednisolone-equivalent dose), total duration of exposure over the past 2 years and date of most recent exposure to oral GCs. Self-reported GC use in the past 24 hours was used to define current use and current dose of oral GCs. Prescription data were used to define the total duration of exposure to oral GCs in the past 2 years—details are given in online supplementary file 3. After comparing current dose reported in the diaries with current dose estimated from prescription data, the measurement error in the prescription data was found to be high. As this measurement error introduces a risk of misclassification bias, measures of historical GC dose derived from CPRD prescription data were not included in the analysis. The most recent exposure to oral GCs was determined for former users of oral GCs. This was defined according to self-report or the prescription data, whichever was most recent.

10.1136/rmdopen-2018-000700.supp3 Supplementary data

### Covariates

The following variables were defined using the EHR: gender, age, Body Mass Index (BMI), socioeconomic status (SES), duration of RA, number of face-to-face general practitioner (GP) visits in the past year,^27^ any prescription for disease-modifying antirheumatic drugs (DMARDs) in the past 2 years and prescriptions for non-oral GCs (including inhaled, injected, topical and other routes) in the past 12 weeks. These variables were defined with reference to the study completion date. SES was defined using the quintile of Townsend score,^28^ which is provided at the patient postcode level by CPRD for the 55% of practices that have agreed to linkage. Duration of RA was calculated from the day the patient first met all the criteria for RA included in the algorithm.^23^ The data preparation steps for defining BMI (kg/m^2^) and exposure to DMARDs (yes/no) are described in online supplementary file 3.

### Analysis

The characteristics of the study population were summarised, with results stratified according to whether or not patients had low MSC, using a minimum cell count of 5. Chi-squared and Kruskal-Wallis tests were performed to investigate possible associations between participant characteristics and low MSC. The prevalence of low MSC was determined and stratified according to current use of oral GCs. Exact CIs were calculated. Due to low numbers, it was not possible to further stratify according to time off GCs; instead, this information is presented graphically. The associations between potential predictors and low MSC were examined using univariate logistic regression, with a significance level of 0.05. Due to the small sample, exact logistic regression^29^ was used unless the model did not converge, in which case the standard large-sample approximation was used.

The main results focused on morning salivary cortisol levels; cortisone levels were also assessed and these results are presented as a sensitivity analysis. The following sensitivity analyses were also performed using information reported by participants in the diaries: the study population was limited by excluding those who reported a major deviation from the sample collection protocol likely to influence the study outcome (eg, taking GCs before collecting the sample) and then excluding those who reported a minor deviation (eg, collecting the sample over 30 min but less than 90 min after waking), which could potentially have influenced the study outcome (details in online supplementary file 4).

10.1136/rmdopen-2018-000700.supp4 Supplementary data

All data cleaning and analyses were performed using Stata/MP V.13.1 (StataCorp LP, College Station, Texas, USA).

## Results

### Main results

Invitations were sent to 526 patients, of whom 117 consented to take part in the study. The full flow of participants has been described previously^21^ and is shown in online supplementary file 5. Of the 117 participants who returned consent forms, 86 returned both their saliva samples and diaries. Of these 86 participants, three saliva samples were inadequate for analysis, one participant did not provide a study completion date and six participants did not have complete follow-up within CPRD at their study completion date. The final study population therefore included 76 participants.

10.1136/rmdopen-2018-000700.supp5 Supplementary data

The characteristics of participants completing the study were similar to those originally listed as eligible in terms of age and gender, although those completing the study tended to be of higher SES, were more likely to have been prescribed DMARDs recently and visited their GP more frequently (online supplementary file 4, table S4.1). The characteristics of the study population are shown in table 1. Half of the participants (n=38) were current GC users. The majority of participants were women (71%) and the median age of the study population was 68.5 years. For the study population, the median (IQR) MSC was 6.5 (2.9–10.8) nmol/L. Overall, 29 of the 76 participants (38%, 95% CI 27% to 50%) had low MSC. Compared with those with normal MSC levels, those with low MSC were older (69 compared with 65 years) and had a longer duration of RA (9.6 compared with 5 years) at the study completion date.

**Table 1:** Characteristics of all participants on the study completion date stratified by normal/low morning salivary cortisol level

	All	Normal MSC	Low MSC	Statistic
n (% total)	76	47 (62% of 76)	29 (38% of 76)	
Female, n (%)	54 (71%)	37 (79%)	17 (59%)	Chi2(1)=3.52, p=0.060
Age (years), median (IQR)	69 (60–75)	65 (58–73)	69 (65–79)	KW(1)=5.14, p=0.023
BMI, median (IQR)	27.9 (23.7–31.2)	28.3 (23.3–34.9)	26.7 (24.1–29.6)	KW(1)=1.10, p=0.295
Townsend score quintile				
1	18%	15%	22%	
2	35%	39%	30%	
3	24%	20%	30%	
4 or 5*	24%	27%	19%	Chi2(3)=2.12, p=0.547
RA duration (years), median (IQR)	7.5 (2.8–10.2)	5.0 (2.3–8.9)	9.6 (7.7–12.2)	KW(1)=9.72, p=0.002
GP visits†, median (IQR)	2 (1–5)	2 (1–5)	3 (2–7)	KW(1)=1.38, p=0.233
DMARD prescription‡	86%	87%	83%	Chi2(1)=0.29, p=0.590
Current oral GC user (%)	50%	28%	86%	Chi2(1)=24.59, p=0.000
Current oral GC dose (mg), median (IQR)	1 (0–5)	0 (0–3)	5 (5–10)	KW(1)=29.10, p=0.0001
Total time exposed to oral GCs‡ (weeks), median (IQR)	50.6 (8.9–101.3)	23.9 (4.9–78.9)	97.4 (52.1–104.4)	KW(1)=10.64, p=0.001
Non-oral GC in past 12 weeks (%)	36%	30%	45%	Chi2(1)=1.77, p=0.183

Eight participants were missing Townsend score, eight participants were missing BMI.

*Minimum cell count=5.

†In past year.

‡In past 2 years.

BMI, Body Mass Index; Chi2, chi-squared; DMARD, disease-modifying antirheumatic drug; GC, glucocorticoid; GP, general practitioner; KW, Kruskal-Wallis; MSC, morning salivary cortisol level; n, number; RA, rheumatoid arthritis.

**Figure 1:**
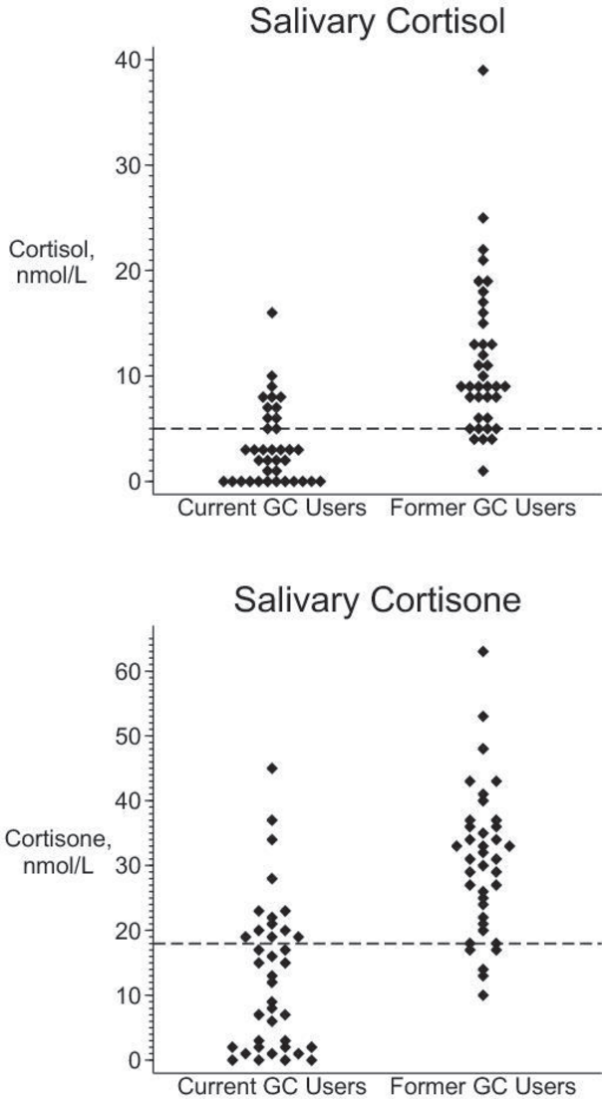
Morning salivary cortisol and cortisone values for current and former users of oral glucocorticoids. GC, glucocorticoid. The dotted lines represent the cut-off values used to indicate low salivary cortisol (5 nmol/L) and cortisone (18 nmol/L). Two extreme values are omitted from the top chart: cortisol levels of 99 and 107 nmol/L (both current users).

**Figure 2:**
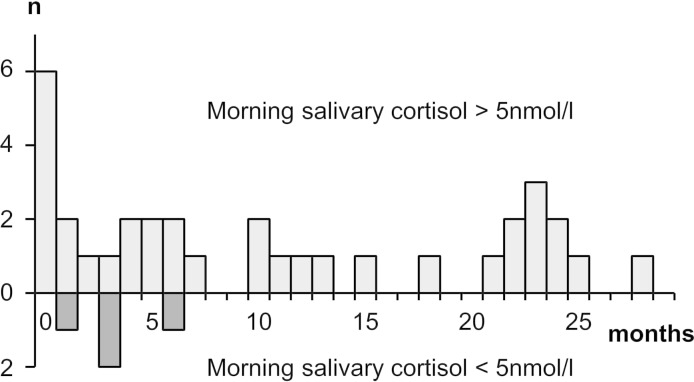
Number of months since last oral glucocorticoid exposure according to morning salivary cortisol level. n, number of participants. Pale grey bars above the axis represent those with normal morning salivary cortisol (>5 nmol/L); dark grey bars below the axis represent those with low morning salivary cortisol (<5 nmol/L). The bar height represents the number of participants (maximum 6).

The MSC for current and former GC users are displayed in figure 1. The median (IQR) MSC for current oral GC users was 3.2 (0–6.5) nmol/L and for former oral GC users was 9.9 (6.6–16.5) nmol/L. Of the 38 current oral GC users, 25 had low MSC giving a prevalence of 66% (95% CI 49% to 80%). Of the 38 participants not currently taking oral GCs, four had low MSC giving a prevalence of 11% (95% CI 3% to 25%). In former users of oral GCs, the longest time since last oral GC exposure was 28 months. For former users with low MSC, the longest time since last oral GC exposure was 6 months (figure 2). There were 18 participants with last oral GC exposure more than 6 months earlier, none of whom had MSC <5 nmol/L. Prescriptions for other GC formulations were examined: 15 of the 38 former oral GC users had a prescription for a non-oral GC in the past 3 months. This included the four participants with low MSC after stopping oral GCs.

Among current users of oral GCs, there was evidence of low MSC at all doses: 3 of 10 (30%) participants taking less than 5 mg prednisolone/day, 14 of 19 (74%) participants taking 5–10 mg prednisolone/day and 8 of 9 (89%) participants taking at least 10 mg prednisolone/day had low MSC.

Current use of oral GCs was strongly associated with low MSC (univariate OR 15.6, 95% CI 4.29 to 74.0). In the whole study population, current dose of oral GCs and cumulative duration of oral GCs were significantly associated with increased risk of low MSC (OR 1.67, 95% CI 1.35 to 2.14 per mg prednisolone equivalent dosage and 1.09, 95% CI 1.04 to 1.15 per month’s exposure, respectively) (table 2). However, when only current oral GC users were included, cumulative duration of oral GCs was no longer associated with low MSC (OR 1.00, 95% CI 0.91 to 1.10). Increasing age and duration of RA were associated with increased risk of low MSC.

**Table 2:** Univariate logistic regression analyses for association between potential risk factors and low morning salivary cortisol

	All participants (n=76)	Current GC users (n=38)
	OR (95% CI)	OR (95% CI)
Current oral GC use	15.62 (4.29 to 73.99)	–
Current oral GC dose (mg)	1.67 (1.35 to 2.14)	1.8 (1.18 to 3.3)
Gender (male vs female)	2.58 (0.84 to 8.17)	3.01 (0.48 to 33.95)
Age (decades)	1.6 (1.05 to 2.54)	1.89 (1.08 to 3.7)
Time exposed to oral GCs in past 2 years (months)	1.09 (1.04 to 1.15)	1.00 (0.91 to 1.10)
Duration of RA (years)*	1.12 (1.02 to 1.23)	1.42 (1.11 to 1.81)

The results shown are univariate ORs for the listed variables for all participants and for current oral GC users only. Moreover, 29 of all 76 participants and 25 of 38 current GC users had low morning salivary cortisol. Months are 28 days.

*Used large sample approximation.

GC glucocorticoid; n, number of participants; OR, odds ratio; CI, confidence interval; RA, rheumatoid arthritis.

### Sensitivity analyses

The salivary cortisone measurements are displayed in figure 1. Comparing low MSC to low morning salivary *cortisone*, seven participants were classified differently. Four participants with a normal MSC had morning salivary cortisone <18 nmol/L (including one additional former GC user), and three participants with a low MSC had salivary cortisone >18 nmol/L. The prevalence estimates of those at risk of AI were similar to the main study results when the sample was restricted to patients reporting no problems following the sample collection procedure (online supplementary file 4, table S4.3). The pattern of increasing prevalence by current dose category was repeated in the sensitivity analyses (online supplementary file 4, table S4.4). In the regression models using low morning cortisone level as the outcome, the OR for current dose was increased (OR 6.05, 95% CI 1.77 to 67.5) and the OR for age was attenuated, becoming non-significant (OR 1.47, 95% CI 0.88 to 2.6). In the remaining sensitivity analyses, the OR for age was attenuated, but the remaining results were similar (online supplementary file 4, table S4.5).

## Discussion

In this group of patients with RA currently or formerly exposed to oral GCs, 29 of 76 (38%) participants had morning salivary cortisol levels below the normal reference range (<5 nmol/L). This included 66% of those currently taking oral GCs and 11% of former oral GC users. Among former users, the maximum time since oral GC withdrawal at which there was evidence of low salivary cortisol levels was 6 months. Current dose of oral GCs was associated with low MSC, whereas total duration of GC exposure was not associated in current users. Increasing age and duration of RA were also associated with low MSC. All participants in this study had received at least one prescription for oral GCs in primary care in the previous 2 years.

Morning salivary cortisol level was used as a surrogate measure of adrenal suppression as this is non-invasive, offers high acceptability to patients and shows very high agreement with the ACTH-stimulation test (sensitivity and specificity above 96%).^19 20^ Therefore, this approach bypasses the need to complete a dynamic test of the HPA axis, such as an ITT or ACTH-stimulation test. However, it is important to note that some studies do show a less robust correlation between MSC and peak stimulated serum cortisol,^30 31^ while others recommend using high and low cut-off values to identify ‘definite’ cases and non-cases, with a grey area between these values.^18^ There is also the possibility that the lower MSC levels in current users compared with former users reflect acute suppression of cortisol by the presence of exogenous GCs. However, the majority of participants reported collecting saliva samples at approximately 24 hours after their last GC dose, which is the typical treatment pause used when assessing HPA-axis function in clinics. The half-life of prednisolone at the doses reported by participants is 3 hours, and the suppressive effects of prednisolone on the HPA axis last approximately 12–24 hours.^32^ With respect to both these concerns, the results of the current study can be compared with a recent study by Borresen *et al*,^33^ which also investigated AI in patients with RA exposed to oral GCs, and a study by Jamilloux *et al*,^16^ which investigated patients with giant cell arteritis. Both of these studies used ACTH-stimulation tests to define AI, with a GC pause of 24 (Jamilloux *et al*) or 48 hours (Borresen *et al*) before testing. These two studies had very similar prevalence estimates of 48% and 49%, which suggests first that there is little difference between a GC gap of 24 versus 48 hours, but second that our estimate of 66% could be an overestimate. On the other hand, both papers included only patients taking 5 mg prednisolone/day whereas our study included patients taking higher doses.

The current literature on adrenal recovery over time is sparse, with very few patients followed up for a significant length of time.^13^ This study adds evidence for suppressed adrenal function after cessation of oral GCs. However, it is possible that the former GC users with low MSC were exposed to other forms of GCs (eg, inhaled GCs) at the time of sampling as all of these patients had a prescription for non-oral GCs within the past 3 months. It is recognised that non-systemic GCs can suppress the HPA axis.^14^ A longitudinal study properly accounting for exposure to any GCs would be the most appropriate means to assess adrenal recovery.

In our study, current GC dose (prednisolone or equivalent in milligrams) was positively associated with low MSC, and this association persisted when only current GC users were considered. Our findings are in agreement with a meta-analysis that demonstrated a trend of increasing AI prevalence from low to high GC dose.^14^ However, our current study found no association with duration of exposure to GCs when only current GC users were considered. In the current study, 75% of current GCs users had active prescriptions covering at least 75% of the 2-year look-back window (online supplementary file 4, table S4.6); therefore, the range of treatment durations may have been too narrow to detect an effect in this small sample of participants. In addition, total duration of GC exposure was defined using prescription data. By comparing prescription data to self-report, we found current GC exposure status was misclassified in approximately 14% of patients (*paper under review*); misclassification may therefore have introduced bias towards the null.

The study had a number of limitations. First, the study sample was small and represents only 2**%** of the potentially eligible patients originally identified within the EHR. Those who took part in the study are likely to differ systematically from those who did not. Indeed, the participants tended to have a higher SES, were more likely to have been prescribed DMARDs and visited their GP more frequently (online supplementary file 4, table S4.1). However, it is unlikely that participants selected into the study based on their association between GC therapy and AI. Second, there is the potential for measurement error and hence a risk of misclassification bias. The choice of study outcome (low MSC) and risk of errors in using prescription data have been discussed elsewhere. It is also possible there were errors in self-reported GC exposure. However, the diary was designed to minimise the risk of reporting errors: participants reported GC use within the past 24 hours, were assured that the information would not be shared with their doctors, and were given instructions, examples and a list of medications of interest to help them complete the diary. Third, as this was a cross-sectional study, cortisol levels before exposure to GCs are unknown and the low MSC may not be attributable to GC exposure. However, participants with potential HPA-axis suppression unrelated to AI were excluded from the study population, and the rate of non-iatrogenic AI in the general population is very low.^10^ There is some evidence suggesting HPA-axis function is altered in patients with RA,^34 35^ although there are conflicting results about whether baseline cortisol levels are raised,^36^ unchanged or reduced^35^ in patients with RA. It is possible that unmeasured features of RA (eg, disease activity or severity) may confound the relationship between GC exposure and cortisol levels. However, whether low MSC was a direct result of GC exposure or not, the high proportion of those patients currently taking GCs who had low MSC remains important. Finally, as the study aimed to investigate adrenal function in RA, no control group was included. Given the potential alterations in HPA-axis function in RA, these results may not be generalisable beyond this patient population.

The current literature on GC-induced adrenal insufficiency is heterogeneous and inconclusive.^13 14^ This study makes several useful contributions: first, although still small, it is larger than most existing studies, with greater power to investigate associations with risk factors. Second, as the literature currently lacks studies into adrenal recovery after withdrawal of GCs,^13^ we specifically recruited former users, measuring MSC levels in those previously exposed to oral GCs. This study demonstrated that low MSC was not seen in patients exposed more than 6 months ago. This gives some clue as to recovery times, although as described above a longitudinal study would be the best means to investigate this. Finally, our study population was homogeneous, with the same GC indication, thus the results are potentially more informative for the target population than existing studies, given the influence of indication on risk of adrenal suppression.^14^

There are no agreed criteria for assessing adrenal suppression in patients treated with therapeutic GCs, with different centres employing different protocols. Based on our current work, we suggest that clinical suspicion should remain high in any patients with possible symptoms even after the immediate post-treatment period is passed. Our study was not designed to test protocols for weaning patients off oral prednisolone and there remain no good evidence-based recommendations. Nonetheless, as the risk of significant adrenal suppression increases with dose and, potentially, duration of treatment, we suggest that patients treated with prednisolone equivalent >10 mg daily for more than 4 weeks be targeted for tapered withdrawal. We suggest rapid reduction in daily dose to prednisolone equivalent 5 mg daily (balanced by clinical need) and then reduce at monthly intervals by 1 mg per day. One month after stopping prednisolone, consider ACTH-stimulation test to verify restoration of adrenal function. Similarly, there is a lack of consensus guidelines in advising patients found to be subject to adrenal suppression. In this regard, a pragmatic approach would be to advise patients of typical symptoms of adrenal suppression, provide them with an information leaflet and arrange definitive testing with an adrenal stimulation test, for example, the ACTH-stimulation test, at an interval, to determine if adrenal function has recovered. However, the evidence base for these suggestions is not robust, and no trials are underway. This is an important area of clinical uncertainty, and identifies a deficiency that should be addressed.

In conclusion, the proportion of current GC users with low morning salivary cortisol levels was high (66%) and included some patients taking doses of less than 5 mg prednisolone/day. A small number of patients (11%) no longer taking oral GCs were found to have low MSC, and the time to recovery remains unclear: some patients who last used GCs 6 months ago had low MSC. As low MSC can indicate AI, was common among current users and occurred even when taking low doses, clinicians should remain vigilant whenever GCs are withdrawn.
