# Supplementary material for: Low salivary cortisol levels in patients with rheumatoid arthritis exposed to oral glucocorticoids: a cross-sectional study set within UK electronic health records
Source: RMD Open. 2018 Oct 1;4(2):e000700. doi: 10.1136/rmdopen-2018-000700 (PMC6173262; doi:10.1136/rmdopen-2018-000700)
Supplement: Supplementary data [file rmdopen-2018-000700supp001.pdf]

Supplementary File 1 List of Read codes used to exclude patients

readcode	desc
43C3.11	HIV positive
7100	Excision of pituitary gland
7100.11	Hypophysectomy operations
7101	Destruction of pituitary gland
7102	Other operations on pituitary gland
7122	Excision of adrenal gland operations
7122.11	Adrenalectomy operations
7123	Operations on aberrant adrenal tissue
7124	Other operations on adrenal gland
A176.00	Tuberculosis of adrenal glands - Addison's disease
A363.00	Waterhouse-Friderichsen syndrome
A788.00	Acquired immune deficiency syndrome
A788.11	Human immunodeficiency virus infection
A788000	Acute human immunodeficiency virus infection
A788100	Asymptomatic human immunodeficiency virus infection
A788200	HIV infection with persistent generalised lymphadenopathy
A788300	Human immunodeficiency virus with constitutional disease
A788400	Human immunodeficiency virus with neurological disease
A788500	Human immunodeficiency virus with secondary infection
A788600	Human immunodeficiency virus with secondary cancers
A788U00	HIV disease result/haematological+immunologic abnorms,NEC
A788W00	HIV disease resulting in unspecified malignant neoplasm
A788X00	HIV disease resulting/unspcf infectious+parasitic disease
A788y00	Human immunodeficiency virus with other clinical findings
A788z00	Acquired human immunodeficiency virus infection syndrome NOS
A789.00	Human immunodef virus resulting in other disease
A789000	HIV disease resulting in mycobacterial infection
A789100	HIV disease resulting in cytomegaloviral disease
A789200	HIV disease resulting in candidiasis
A789300	HIV disease resulting in Pneumocystis carinii pneumonia
A789311	HIV disease resulting in Pneumocystis jirovecii pneumonia
A789400	HIV disease resulting in multiple infections
A789500	HIV disease resulting in Kaposi's sarcoma
A789600	HIV disease resulting in Burkitt's lymphoma
A789700	HIV dis resulting oth types of non-Hodgkin's lymphoma
A789800	HIV disease resulting in multiple malignant neoplasms
A789900	HIV disease resulting in lymphoid interstitial pneumonitis
A789A00	HIV disease resulting in wasting syndrome
A789X00	HIV dis reslt/oth mal neopl/lymph,h'matopoetc+reltd tissu
B540.00	Malignant neoplasm of adrenal gland
B540.11	Phaeochromocytoma
B540000	Malignant neoplasm of adrenal cortex
B542.00	Malignant neoplasm pituitary gland and craniopharyngeal duct

B542000	Malignant neoplasm of pituitary gland
B587.00	Secondary malignant neoplasm of adrenal gland
B7H0.00	Benign neoplasm of adrenal gland
B7H2.00	Benign neoplasm of pituitary gland and craniopharyngeal duct
B7H2.11	Pituitary adenoma
B7H2000	Benign neoplasm of pituitary gland
B8yy100	Carcinoma in situ of adrenal gland
B8yy300	Carcinoma in situ of pituitary gland
B920.00	Neop uncertain behaviour pituitary and craniopharyngeal duct
B920000	Neoplasm of uncertain behaviour of pituitary gland
B922.00	Neoplasm of uncertain behaviour of adrenal gland
C13..00	Disorders of pituitary gland and its hypothalamic control
C13..11	Hypothalamus disorders
C131.00	Other anterior pituitary hyperfunction
C131.11	Forbes - Albright syndrome
C132.00	Panhypopituitarism
C132.11	Hypopituitarism NOS
C132.12	Sheehan's syndrome
C132.13	Simmond's disease
C132000	Idiopathic panhypopituitarism
C132100	Post-birth injury panhypopituitarism
C132200	Postinfarction panhypopituitarism
C132300	Postinfective panhypopituitarism
C132y00	Other specified panhypopituitarism
C132z00	Panhypopituitarism NOS
C134400	Isolated ACTH deficiency
C134411	ACTH deficiency
C137100	Post-hypophysectomy hypopituitarism
C137111	Surgically-induced hypopituitarism
C137200	Post-radiotherapy hypopituitarism
C150000	Idiopathic Cushing's syndrome
C150200	Pituitary dependent Cushing's syndrome
C150300	Ectopic ACTH secretion causing Cushing's syndrome
C150400	Nelson's syndrome
C150500	Alcohol-induced pseudo-Cushing's syndrome
C152812	Congenital adrenal hyperplasia NEC
C152813	Congenital adrenal gland hypertrophy NEC
C153.00	Other corticoadrenal overactivity
C153.11	ACTH overproduction
C153.12	Adrenocortical hyperfunction
C154200	Adrenal haemorrhage
C154211	Adrenocortical haemorrhage
C154500	Postprocedural adrenocortical(-medullary) hypofunction
C18..00	Polyglandular dysfunction and related disorders
C180.00	Multiple endocrine adenomatosis
C180.11	Wermer's syndrome

C181.00	Other combinations of endocrine dysfunction
C181.11	Lloyd's syndrome
C181.12	Schmidt's syndrome
C182.00	Autoimmune polyglandular failure
C183.00	Polyglandular hyperfunction
C18y.00	Other specified polyglandular dysfunction
C18z.00	Polyglandular dysfunction NOS
C308.00	Disorders of fatty-acid metabolism
