# Supplementary material for: Low salivary cortisol levels in patients with rheumatoid arthritis exposed to oral glucocorticoids: a cross-sectional study set within UK electronic health records
Source: RMD Open. 2018 Oct 1;4(2):e000700. doi: 10.1136/rmdopen-2018-000700 (PMC6173262; doi:10.1136/rmdopen-2018-000700)
Supplement: Supplementary data [file rmdopen-2018-000700supp003.pdf]

Supplementary File 3 Definition of variables using CPRD datasets

3.1 Exposure to oral glucocorticoids / disease-modifying anti-rheumatic drugs

An algorithm, coded using Stata, was used to define current use of these medications from the CPRD prescription data. The decisions taken are summarised in the flow diagram below. The algorithm can be shared by contacting the authors.

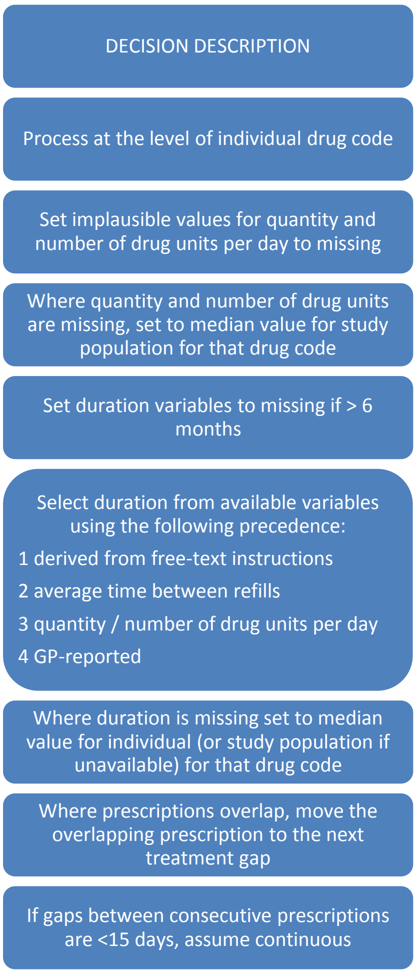

3.2 Defining body mass index

1. Clean height: set to missing if <1.21m, >2.14m or recorded prior to age 18
2. Calculate median height for each patient
3. Clean weight: set to missing if <20kg or >450kg
4. Clean weight: identify further outliers by fitting a random intercepts model (adjusting for age and gender) regressing weight on time (grouping: patient) and calculating standardised residuals. Set to missing
5. Drop weights recorded more than 5 years prior to study completion date
6. Keep only the weight recorded closest to the study completion date
7. Calculate BMI (weight / (height*height))

Defining BMI within the Clinical Practice Research Datalink. RA rheumatoid arthritis, BMI body mass index. Method developed by Ruth Costello, ARUK Centre for Epidemiology, The University of Manchester.

Figure reproduced from:

Joseph, R. M., Movahedi, M., Dixon, W. G. and Symmons, D. P. M. (2016), Smoking-Related Mortality in Patients With Early Rheumatoid Arthritis: A Retrospective Cohort Study Using the Clinical Practice Research Datalink. Arthritis Care & Research, 68: 1598–1606. doi:10.1002/acr.22882

This article is available under the terms of the Creative Commons Attribution License (CC BY) (which may be updated from time to time) and permits use, distribution and reproduction in any medium, provided that the Contribution is properly cited.
