# Supplementary material for: Low salivary cortisol levels in patients with rheumatoid arthritis exposed to oral glucocorticoids: a cross-sectional study set within UK electronic health records
Source: RMD Open. 2018 Oct 1;4(2):e000700. doi: 10.1136/rmdopen-2018-000700 (PMC6173262; doi:10.1136/rmdopen-2018-000700)
Supplement: Supplementary data [file rmdopen-2018-000700supp004.pdf]

Supplementary File 4) Additional Results

Contents

Part 1 Characteristics of patients not included in analysis 1

Table S4.1 Patient characteristics by stage of recruitment 1

Part 2 Sensitivity analyses 2

Description of sensitivity analyses 2

Table S4.2 Distribution of morning salivary cortisol and morning salivary cortisone by current GC use status 2

Table S4.3 Prevalence of low salivary cortisol/cortisone by current oral GC use status sensitivity analyses 3

Table S4.4 Prevalence of low salivary cortisol/cortisone by current oral GC dose (current users only) sensitivity analyses 3

Table S4.5 Univariate logistic regression for risk of low salivary cortisol/cortisone, sensitivity analysis 4

Part 3 Characteristics according to current oral glucocorticoid GC use status 5

Table S4.6 Participant characteristics on study completion date according to current use of oral GCs 5

Part 1 Characteristics of patients not included in analysis

Table S4.1 Patient characteristics by stage of recruitment

n number; IQR inter-quartile range, DMARDs disease-modifying anti-rheumatic drugs; GP general practice; CPRD Clinical Practice Research Datalink

*in the year prior to 30/11/2015

Sub-population	Number of participants	Female, n (%)	Age (years), median (IQR)	Townsend score quintile, median (IQR)	DMARDs - ever*, n (%)	GP visits* (n), median (IQR)
All eligible patients within CPRD	3718	2640 (71%)	68 (58-77)	3 (2-4)	2348 (63.2%)	2 (0-4)
Practice agreed to mail-out	761	535 (70.3%)	69 (59-77)	3 (2-4)	566 (74.4%)	3 (2-6)
Patient recruited to study	117	84 (71.8%)	69 (60-74)	3 (2-3)	92 (78.6%)	4 (2-6)
Participant returned sample and diary	86	60 (69.8%)	68.5 (60-74)	2 (2-3)	67 (77.9%)	3 (1-6)

Part 2 Sensitivity analyses

Description of sensitivity analyses

The following sensitivity analyses were performed:

<u>Outcome definition</u>

- Main outcome: morning cortisol <5nmol/l
- Secondary outcome: morning cortisone <18nmol/l

<u>Sampling process</u>

Four Instructions to collect samples correctly:
1) Collect sample 15-30 minutes after waking
2) Collect samples on a "rest" day when wake without an alarm
3) Don't eat, drink, smoke or brush teeth until after sampling
4) Don't take any GCs until after sampling

Three categories for quality of sampling:
1 (no problems) meets 1)- 4)
2 (minor problems) sample collection outside 1) but still <90 mins; or 'no' for ALL of 3)
3 (major problems) failed 1), 2), 3) OR 4), except as above; missing for any of 1)-4)

Analyses:

- All participants, regardless of any reported sampling problems
- Only participants with no major sampling problems reported
- Only participants with no sampling problems (major OR minor) reported

Table S4.2 Distribution of morning salivary cortisol and morning salivary cortisone by current GC use status

n number; GC glucocorticoid; IQR interquartile range

	All participants (n=76)	Former GC users (n=38)	Current GC users (n=38)
Cortisol, median (IQR)	6.5 (2.9-10.8) nmol/L	9.9 (6.6-16.5) nmol/L	3.2 (0-6.5) nmol/L
Cortisone, median (IQR)	21.2 (1.8-33.4) nmol/L	31.4 (22.3-36.6) nmol/L	13.2 (2.7-20.5) nmol/L

Table S4.3 Prevalence of low salivary cortisol/cortisone by current oral GC use status sensitivity analyses

GC glucocorticoid; CI confidence interval

Results shown are number of participants with the outcome/ number of participants in group, prevalence of outcome in that group, and 95% confidence intervals.

Main outcome	All participants	Former GC users	Current GC users
All participants	29/76; prevalence 38% (95% CI 27%-50%)	4/38; prevalence 11% (95% CI 3%-25%)	25/38; prevalence 66% (95% CI 49%-80%)
No major sampling problems	28/68; prevalence 41% (95% CI 29%-54%)	4/33; prevalence 12% (95% CI 3%-28%)	24/35; prevalence 69% (95% CI 51%-83%)
No major or minor sampling problems	21/48; prevalence 44% (95% CI 29%-59%)	3/21; prevalence 14% (95% CI 3%-36%)	18/27; prevalence 67% (95% CI 46%-83%)
Secondary outcome	All participants	Former GC users	Current GC users
All participants	30/76; prevalence 39% (95% CI 28%-51%)	5/38; prevalence 13% (95% CI 4%-28%)	25/38; prevalence 66% (95% CI 49%-80%)
No major sampling problems	29/68; prevalence 43% (95% CI 31%-55%)	5/33; prevalence 15% (95% CI 5%-32%)	24/35; prevalence 69% (95% CI 51%-83%)
No major or minor sampling problems	21/48; prevalence 44% (95% CI 29%-59%)	3/21; prevalence 14% (95% CI 3%-36%)	18/27; prevalence 67% (95% CI 46%-83%)

Table S4.4 Prevalence of low salivary cortisol/cortisone by current oral GC dose (current users only) sensitivity analyses

GC glucocorticoid; CI confidence interval

Results shown are number of participants with the outcome/ number of participants in group, prevalence of outcome in that group, and 95% confidence intervals.

Main outcome	dose <5mg	dose 5-10mg	dose>=10mg
All participants	3/10; prevalence 30% (95% CI -5%-65%)	14/19; prevalence 74% (95% CI 52%-95%)	8/9; prevalence 89% (95% CI 63%-115%)
No major sampling problems	3/9; prevalence 33% (95% CI -5%-72%)	13/17; prevalence 76% (95% CI 54%-99%)	8/9; prevalence 89% (95% CI 63%-115%)
No major or minor sampling problems	3/9; prevalence 33% (95% CI -5%-72%)	10/13; prevalence 77% (95% CI 50%-103%)	5/5; prevalence 100% (95% CI 100%-100%)
Secondary outcome	dose <5mg	dose 5-10mg	dose>=10mg
All participants	2/10; prevalence 20% (95% CI -10%-50%)	14/19; prevalence 74% (95% CI 52%-95%)	9/9; prevalence 100% (95% CI 100%-100%)
No major sampling problems	2/9; prevalence 22% (95% CI -12%-56%)	13/17; prevalence 76% (95% CI 54%-99%)	9/9; prevalence 100% (95% CI 100%-100%)
No major or minor sampling problems	2/9; prevalence 22% (95% CI -12%-56%)	11/13; prevalence 85% (95% CI 62%-107%)	5/5; prevalence 100% (95% CI 100%-100%)

Table S4.5 Univariate logistic regression for risk of low salivary cortisol/cortisone, sensitivity analysis

CI confidence interval; GC glucocorticoid

The results shown are univariate odds ratios for the listed variables for all participants and for current users only.

*Results for exact logistic regression, except for * which are standard large-sample approximation*

	MAIN OUTCOME		SECONDARY OUTCOME	
**All participants**	All participants<br>Odds Ratio (95% CI)	Current GC users<br>Odds Ratio (95% CI)	All participants<br>Odds Ratio (95% CI)	Current GC Users<br>Odds Ratio (95% CI)
Current oral GC use	15.62 (4.29 - 73.99)	--	12.19 (3.6 - 49.92)	--
Current oral GC dose (mg)	1.67 (1.35 - 2.14)	1.8 (1.18 - 3.3)	1.75 (1.4 - 2.28)	6.05 (1.77 - 67.45)
Gender (male vs. female)	2.58 (.84 - 8.17)	3.01 (.48 - 33.95)	2.37 (.77 - 7.47)	3.01 (.48 - 33.95)
Age (10 years)	1.6 (1.05 - 2.54)	1.89 (1.08 - 3.7)	1.42 (.96 - 2.18)	1.47 (.88 - 2.6)
Time exposed to oral GCs, past 2 years (4 weeks)	1.09 (1.04 - 1.15)	1 (.91 - 1.1)	1.08 (1.03 - 1.14)	1.02 (.93 - 1.11)
Duration of RA (years)*	1.12 (1.02 - 1.23)	1.42 (1.11 - 1.81)	1.1 (1.01 - 1.21)	1.28 (1.04 - 1.57)
**No major sampling problems**	All participants<br>Odds Ratio (95% CI)	Current GC Users<br>Odds Ratio (95% CI)	All participants<br>Odds Ratio (95% CI)	Current GC Users<br>Odds Ratio (95% CI)
Current oral GC use	15.03 (3.97 - 73.63)	--	11.68 (3.31 - 49.7)	--
Current oral GC dose (mg)	1.65 (1.33 - 2.13)	1.76 (1.16 - 3.2)	1.73 (1.38 - 2.29)	5.66 (1.71 - 62.26)
Gender (male vs. female)	2.95 (.9 - 10.21)	2.63 (.4 - 30.41)	2.69 (.83 - 9.27)	2.63 (.4 - 30.41)
Age (10 years)	1.53 (.98 - 2.47)	1.84 (.99 - 3.81)	1.34 (.88 - 2.1)	1.36 (.77 - 2.52)
Time exposed to oral GCs, past 2 years (4 weeks)	1.08 (1.03 - 1.14)	.97 (.86 - 1.08)	1.07 (1.02 - 1.13)	1 (.89 - 1.1)
Duration of RA (years)*	1.1 (1 - 1.21)	1.51 (1.12 - 2.03)	1.09 (.99 - 1.19)	1.32 (1.04 - 1.67)
**No major or minor sampling problems**	All participants<br>Odds Ratio (95% CI)	Current GC Users<br>Odds Ratio (95% CI)	All participants<br>Odds Ratio (95% CI)	Odds Ratio (95% CI)
Current oral GC use	11.29 (2.41 - 75.75)	--	11.29 (2.41 - 75.75)	--
Current oral GC dose (mg)	1.8 (1.35 - 2.6)	3.65 (1.42 - 16.86)	1.88 (1.38 - 2.78)	8.28 (1.8 - 154.98)
Gender (male vs. female)	6.95 (1.42 - 47.15)	4.83 (.46 - 257.59)	6.95 (1.42 - 47.15)	4.83 (.46 - 257.59)
Age (10 years)	1.32 (.77 - 2.32)	1.55 (.83 - 3.12)	1.3 (.76 - 2.28)	1.52 (.82 - 3.03)
Time exposed to oral GCs, past 2 years (4 weeks)	1.09 (1.03 - 1.17)	.98 (.85 - 1.11)	1.1 (1.03 - 1.17)	.99 (.86 - 1.11)
Duration of RA (years)*	1.11 (.99 - 1.25)	1.58 (1.1 - 2.26)	1.08 (.97 - 1.2)	1.27 (.98 - 1.65)

Part 3 Characteristics according to current oral glucocorticoid GC use status

Table S4.6 Participant characteristics on study completion date according to current use of oral GCs

n number; IQR interquartile range; Chi2 chi-squared test; BMI body mass index; KW Kruskal-Wallis test; RA rheumatoid arthritis; GP general practice; DMARD disease-modifying anti-rheumatid drugs; GC glucocorticoid

Results shown for all participants, former users of oral GCs and current users of oral GCs

*results suppressed for former users as n<5 in some groups

	All	Former User	Current User	Statistic
n (% total)	76	38 (50% of 76)	38 (50% of 76)	
Female, n (%)	54 (71%)	27 (71%)	27 (71%)	Chi2(1)=0.00, p=1.000
Age (years), median (IQR)	69 (60-75)	66 (60-73)	69 (59-78)	KW(1)=1.50, p=0.220
BMI, median (IQR)	27.9 (23.7-31.2)	28.4 (23.9-36.7)	26.7 (23.3-30.2)	KW(1)=2.28, p=0.131
Townsend score quintile*				
1	17.6%		26.5%	
2	35.3%		35.3%	
3	23.5%		20.6%	
4 or 5	23.5%		17.6%	Chi2(3)=4.25, p=0.236
RA duration (years), median (IQR)	7.5 (2.8-10.2)	5.3 (2.3-9.5)	8.5 (4.7-10.8)	KW(1)=2.61, p=0.106
n GP visits in past year, median (IQR)	2 (1-5)	2 (1-5)	2 (2-6)	KW(1)=0.48, p=0.490
DMARD prescription in past 2 years	85.5%	89.5%	81.6%	Chi2(1)=0.96, p=0.328
Current oral GC dose (mg), median (IQR)	1 (0-5)	0 (0-0)	5 (5-8)	KW(1)=56.26, p=0.0001
Total time exposed to oral GCs over past 2 years (weeks), median (IQR)	50.6 (8.9-101.3)	11.4 (4.1-34.3)	98.7 (78.1-104.4)	KW(1)=33.36, p=0.0001
Prescribed non-oral formulation GCs in past 12 weeks (%)	35.5%	39.5%	31.6%	Chi2(1)=0.52, p=0.472
