# Supplementary material for: Low salivary cortisol levels in patients with rheumatoid arthritis exposed to oral glucocorticoids: a cross-sectional study set within UK electronic health records
Source: RMD Open. 2018 Oct 1;4(2):e000700. doi: 10.1136/rmdopen-2018-000700 (PMC6173262; doi:10.1136/rmdopen-2018-000700)

Supplementary File 5 Flow of participants through the study

Reproduced from: Joseph RM, Soames J, Wright M, Sultana K, van Staa TP, Dixon WG. Supplementing electronic health records through sample collection and patient diaries: A study set within a primary care research database. Pharmacoepidemiol Drug Saf. 2017;1–4. https://doi.org/10.1002/pds.4323

This article is available under the terms of the Creative Commons Attribution License (CC BY) (which may be updated from time to time) and permits use, distribution and reproduction in any medium, provided that the Contribution is properly cited.

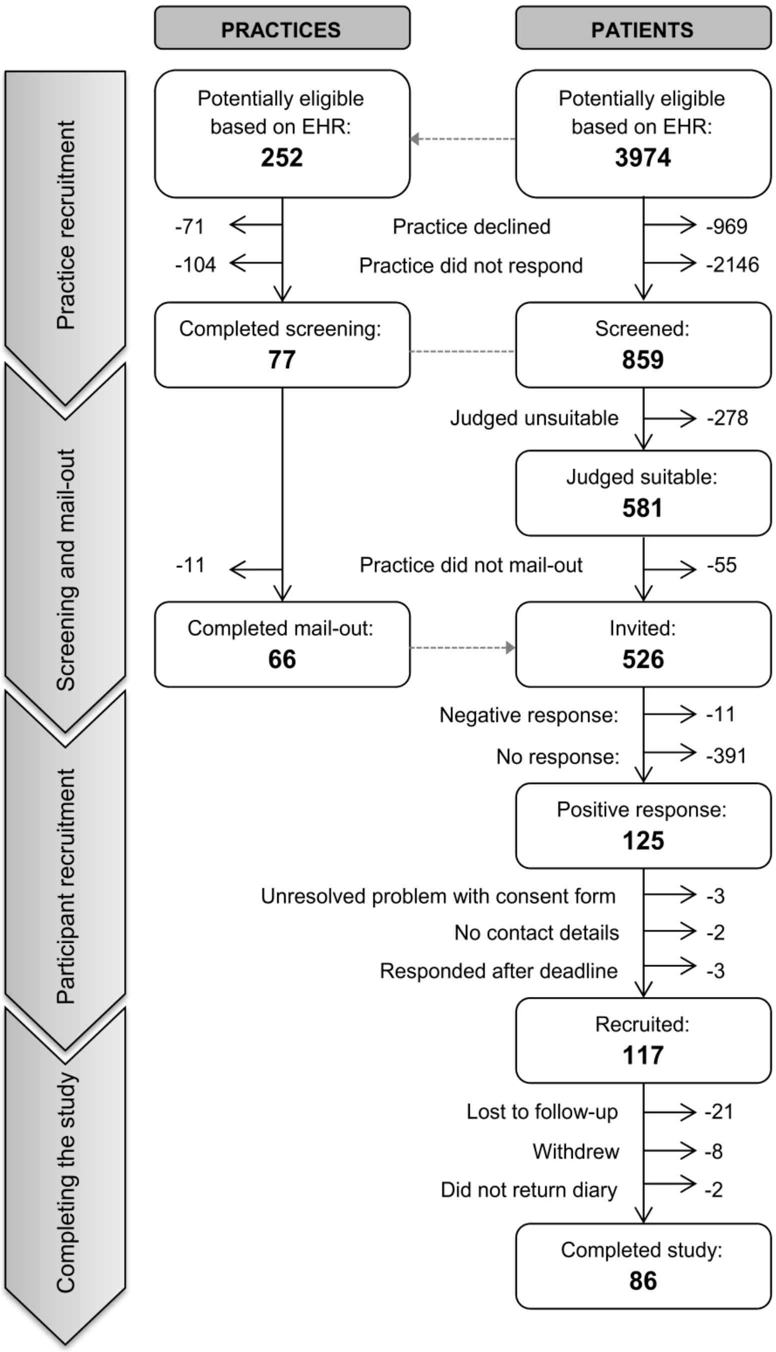

Supplement: Supplementary data [file rmdopen-2018-000700supp005.pdf]
